# Safety of adenosine stress perfusion cardiac MRI in patients undergoing lung transplantation evaluation

**DOI:** 10.1186/1532-429X-18-S1-P104

**Published:** 2016-01-27

**Authors:** Sung A Chang, Marco A Cordeiro, Han W Kim, Michele Parker, Igor Klem, Anna Lisa Crowley, Raymond Kim

**Affiliations:** 1 Cardiology, Duke University Hospital, Durham, NC USA; 2 Radiology, Duke University, Durham, NC USA

## Background

Assessment of coronary artery disease in lung disease patients is frequently challenging because of physical limitations for exercise and poor echocardiographic windows. Vasodilator stress perfusion cardiac magnetic resonance imaging (CMR) does not require physical exercise and may provide good images. However, fear of aggravating bronchoconstriction limits the use of adenosine stress testing in patients with severe lung disease. We are unaware of any published study testing the use and/or safety of adenosine stress testing in patients undergoing lung transplantation evaluation.

## Methods

Patients undergoing stress perfusion CMR for lung transplantation evaluation were consented and enrolled in a prospective CMR registry from February 2006 to April 2015. Patients with asthma or bronchospastic disease who required daily bronchodilator use were not considered for stress testing. Serious adverse events (SAEs) were defined as death, myocardial infarction, or cardiopulmonary instability requiring treatment and stopping the examination. Adverse events (AEs) included SAEs and any transient symptom/signs which did not require stopping the CMR examination.

## Results

271 patients were consecutively enrolled. Mean age was 64 ± 9 years old (84% male). Idiopathic pulmonary fibrosis and chronic obstructive lung disease were the leading cause of lung disease (67% and 18%). Spirometry finding demonstrated that 80% (n = 218) of patients met criteria of moderate or severe restrictive lung disease, and 85% showed moderate or severe obstructive disease. All patients were on home oxygen therapy or supplemental oxygen therapy.

Adverse events were observed in 16 patients (5.9%, 95% CI=3.6\7%-9.5%; see Table 1). There was one severe adverse event (0.36%, 95% CI= 0.01-2.01). This patient had severe dyspnea during adenosine infusion which required early termination of the exam however hypoxemia was not demonstrated (O_2_ saturation less than 90%) and the patient spontaneously recovered after discontinuation of adenosine without further treatment. 95% confidence intervals for SAEs and AEs are displayed in the figure with a comparison to a conventional cohort without lung disease. All completed stress perfusion CMR scans were adequate quality for clinical determination of the presence or absence of ischemic disease.

**Table 1:** Adverse Events During Stress CMR

Adverse Events During Stress CMR (271 patients)	N	%	95% C.I.
Adverse Event			
Any	16	5.9	3.4, 9.4%
Transient heart block	10	3.7	1.8, 6.7%
Paroxysmal atrial fibrillation	1	0.4	0.01, 2.04%
Dyspnea aggravation	5	1.9	0.6, 4.3%
**Serious Adverse Event**			
Any	1	0.4	0.01, 2.04%
Cardiopulmonary instability / requiring treatment or early scan termination	1	0.4	0.01, 2.04%
Death/myocardial infarction	0	0.0	0.00, 1.11%

## Conclusions

This is the first study to evaluate the safety of adenosine stress perfusion CMR in patients with severe lung disease undergoing evaluation for lung transplantation. The frequency of adverse events is similar to that found in previous studies of conventional cohorts without lung disease.

**Figure 1:**
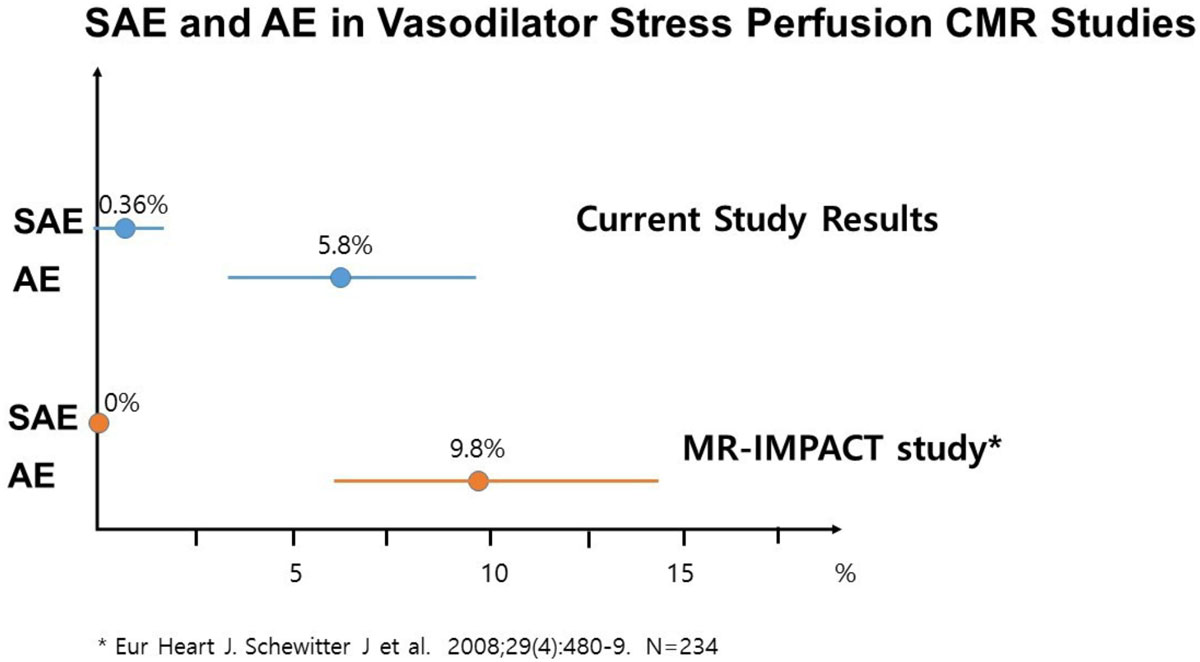
Figure 1

